# Biomarkers for Recurrence and Prognosis in Metastatic Urothelial Cancer: Emerging Clinical Applications

**DOI:** 10.7150/jca.123907

**Published:** 2026-01-01

**Authors:** Alicia Sánchez Cendra, Linda Rocio Ospino, Leonel Pekarek, Yumna Dbouk, Sami Chnaiker, Ana Luengo, Tania Villamor, Amalia Corralo, Raul Diaz-Pedrero, Laura Lopez-Gonzalez, Miguel A Saez, Majd N Michael Alhaddadin, María Belén Alonso-Bartolome, Carlos Casanova-Martín, Melchor Alvarez-Mon, Leonor Garcia Rodriguez, Silvestra Barrena-Blázquez, Miguel A Ortega

**Affiliations:** 1 Oncology Service, University Hospital of Guadalajara, 19002 Guadalajara, Spain.; 2 Department of Medicine and Medical Specialities, Faculty of Medicine and Health Sciences, Network Biomedical Research Center for Liver and Digestive Diseases (CIBEREHD), University of Alcalá, 28801 Alcala de Henares, Spain.; 3 Ramón y Cajal Institute of Sanitary Research (IRYCIS), 28034 Madrid, Spain.; 4 Department of General and Digestive Surgery, Príncipe de Asturias University Hospital, 28805 Alcala de Henares, Spain.; 5 Department of Surgery, Medical and Social Sciences, Faculty of Medicine and Health Sciences, University of Alcalá, 28801 Alcala de Henares, Spain.; 6 Pathological Anatomy Service, Central University Hospital of Defence-UAH Madrid, 28801 Alcala de Henares, Spain.; 7 Immune System Diseases-Rheumatology, Oncology Service and Internal Medicine (CIBEREHD), Príncipe de Asturias University Hospital, 28806 Alcala de Henares, Spain.; 8 Group for Research in Community Care and Social Determinants of Health, University of Alcalá, Madrid, Spain.

**Keywords:** urothelial cancer, biomarkers, prognosis, recurrence, metastatic, liquid biopsy, circulating tumor DNA, circulating tumor cells, FGFR3, microRNA

## Abstract

Urothelial cancer (UC) remains a highly recurrent and heterogeneous malignancy in which reliable biomarkers for recurrence and prognosis are needed, particularly in the metastatic setting. In recent years, the identification and validation of biomarkers have become an essential pillar for improving the diagnosis, monitoring, and prognosis of this disease. This review summarizes and analyzes recent advances in the study of serological, urinary, histological, genetic, and microRNA biomarkers, as well as emerging tools such as circulating tumor cells (CTCs) and circulating tumor DNA (ctDNA). Together, these non-invasive markers show significant potential to complement conventional diagnostic techniques, optimize risk stratification, and support a more personalized therapeutic approach. Furthermore, the integration of new sequencing technologies and liquid biopsy methods is opening new perspectives for the early detection of recurrence and the dynamic assessment of treatment response. However, the routine clinical implementation of these biomarkers still requires validation through standardized prospective studies.

## Introduction

The urinary system comprises the kidneys, ureters, urinary bladder, and urethra, which function together to produce, store, and excrete urine. Most of these structures are lined by urothelium, a specialized transitional epithelium extending from the renal pelvis to the proximal urethra. This epithelial lining acts as a protective barrier against urinary toxins and pathogens while maintaining flexibility during bladder filling and voiding. Urothelial carcinoma can arise from any part of this urothelial tract, although the bladder is by far the most common site. UC is one of the most prevalent malignancies affecting the urinary tract, ranking as the tenth most common cancer worldwide. It accounts for approximately 90-95% of all bladder cancers and is the fourth most common cancer in men and the eighth in women 1. In Europe, its incidence is notably higher in Mediterranean and Western European countries, primarily due to the prevalence of tobacco consumption, the leading risk factor for this disease 2. The global burden of UC is expected to rise due to aging populations and the persistence of environmental and lifestyle-related risk factors 3.

Despite diagnostic and therapeutic advances, UC remains challenging due to its high recurrence and progression rates. At diagnosis, UC is classified into two main categories: non-muscle-invasive bladder cancer (NMIBC), which constitutes up to 80% of cases, and muscle-invasive bladder cancer (MIBC), comprising the remaining 20% 4. Notably, NMIBC has a recurrence rate of up to 50%, with 30% of these cases eventually progressing to MIBC, necessitating more aggressive treatment approaches 5.

The diagnostic process for UC primarily relies on cystoscopy, urinary cytology, and imaging techniques. However, these methods have inherent limitations, including their invasive nature and limited sensitivity, particularly for detecting low-grade tumors 6. Consequently, significant efforts have been dedicated to identifying novel biomarkers that can enhance the accuracy and efficiency of UC diagnosis and prognosis.

Recent research has explored the role of serological, genetic, histological, and molecular biomarkers in UC. Circulating tumor cells (CTCs) and microRNAs (miRNAs) have emerged as promising non-invasive biomarkers with potential applications in early detection, prognosis, and treatment monitoring 7. Liquid biopsy techniques, particularly next-generation sequencing (NGS), have enabled the identification of key genetic alterations, such as FGFR3, TP53, and PIK3CA mutations, which play crucial roles in UC pathogenesis and treatment stratification 8.

Histological classification remains essential for UC management, as variant histological subtypes, including micropapillary, plasmacytoid, and sarcomatoid carcinoma, are associated with distinct prognostic implications and therapeutic responses 9. Additionally, advances in molecular subtyping have provided deeper insights into tumor heterogeneity, guiding more personalized treatment approaches 10.

Despite these advancements, significant challenges persist in the clinical management of UC. The heterogeneity of the disease complicates treatment decisions, and resistance to conventional therapies remains a major obstacle. Immunotherapy, particularly immune checkpoint inhibitors targeting PD-L1, has revolutionized UC treatment, offering durable responses in a subset of patients 11. However, identifying reliable predictive biomarkers for immunotherapy response remains a key area of ongoing research 12.

This review aims to explore the latest advancements in UC biomarkers, including serological, genetic, histological, and molecular markers. It will also discuss the potential of liquid biopsy and NGS in refining diagnosis and treatment strategies, as well as the current challenges and future perspectives in the clinical management of urothelial cancer.

## Serological and Urinary Biomarkers

Serological and urinary biomarkers play a crucial role in the detection, diagnosis, and monitoring of urothelial cancer, providing less invasive alternatives to cystoscopy. These biomarkers can be obtained from blood or urine samples using various methods, including immunocytochemistry, polymerase chain reaction (PCR), and enzyme-linked immunosorbent assays (ELISA). Their sensitivity and specificity vary depending on the marker, with some, such as NMP22 and UroVysion FISH, demonstrating high diagnostic accuracy, particularly in high-grade tumors. However, limitations in precision and the potential for false positives or negatives have prevented their use as standalone diagnostic tools. Instead, they are commonly employed in conjunction with other clinical assessments to improve diagnostic reliability 13,14.

### Serological Biomarkers

Bladder Cancer-Specific Antigen-1 (BLCA-1): BLCA-1 as a protein selectively expressed in bladder cancer cells, making it a promising biomarker for both urinary and serological detection. It becomes detectable in urine and serum following tumor lysis, suggesting its potential utility in non-invasive diagnostic methods 15. Research indicates that BLCA-1 is closely associated with inflammatory cytokines, including vascular endothelial growth factor (VEGF), matrix metalloproteinase-9 (MMP9), interleukin-1 alpha (IL-1α), and interleukin-8 (IL-8), all of which play a role in tumor progression and angiogenesis 15, 16. Despite its potential, further large-scale studies are needed to validate its clinical relevance and standardize its use in diagnostic protocols.

Podoplanin: Podoplanin is a transmembrane glycoprotein involved in cell differentiation, immune response modulation, and tumor progression. It plays a crucial role in epithelial-mesenchymal transition (EMT) and is implicated in the promotion of tumor invasiveness and lymphangiogenesis 17. Sankiewicz et al. 18 reported significantly elevated levels of podoplanin in both plasma and urine samples from patients with aggressive and multifocal bladder tumors. The study demonstrated a diagnostic sensitivity of 72% and specificity of 69%, indicating its potential as a complementary biomarker for bladder cancer detection. However, further research is required to refine its diagnostic thresholds and establish its prognostic value.

Cystatin C: Cystatin C is a low-molecular-weight protein primarily known for its role as an endogenous inhibitor of cysteine proteases (cathepsins), which are crucial in tumor invasion and metastasis. It is also a marker of renal function, as its serum concentration is largely dependent on glomerular filtration rate (GFR). Tokarzewicz et al. 19 found that cystatin C levels were significantly lower in patients with urothelial carcinoma compared to healthy controls, suggesting a possible role in tumor suppression. While it shows promise as a biomarker, its utility in bladder cancer diagnosis remains under investigation due to its strong correlation with renal function.

Aromatase (CYP19A1): Aromatase is an enzyme responsible for the conversion of androgens into estrogens, contributing to the establishment of a tumor-promoting microenvironment. Increased aromatase expression has been observed in the tumor stroma of bladder cancer patients, and it has been associated with higher tumor aggressiveness and reduced overall survival rates 20. Studies suggest that estrogen signaling through aromatase activity may facilitate tumor progression, particularly in muscle-invasive bladder cancer (MIBC). Nguyen et al. 21 highlighted its role in bladder cancer staging and prognosis, emphasizing the need for further investigation into potential therapeutic interventions targeting aromatase in bladder cancer treatment.

### Urinary Biomarkers

CYFRA21-1: A cytokeratin fragment released by urothelial tumor cells. Kuang et al. 22 reported elevated urinary levels in metastatic cases compared to locally invasive disease, highlighting its potential as a prognostic marker.

NMP22: A nuclear mitotic apparatus protein evaluated through immunofluorescence-based urine tests, which enhance the diagnostic accuracy of cystoscopy 23, 24. Its sensitivity ranges from 70% to 70.5%, with specificity varying between 43.2% and 92%. However, factors such as age, benign conditions, and certain medications can lead to false positives 25.

BLCA-4: A nuclear matrix protein excreted in urine with high sensitivity (89-97.37%) and specificity (90-100%). Its overexpression is linked to high-grade tumors and a greater tumor burden, reinforcing its clinical relevance 26.

BTA (Bladder Tumor Antigen): A product of basal membrane degradation by tumor cells. Two urinary assays—BTA-Stat and BTA-Trak—are available for its detection. When used together, these tests reduce false positives associated with hematuria or benign prostatic hyperplasia 26, 27.

Survivin: An apoptosis inhibitor involved in tumor resistance and cell cycle regulation. Its overexpression, particularly in combination with Ki-67, β-catenin, and p53, correlates with poor prognosis and reduced survival rates 26.

BLCA-1: Previously mentioned as a promising diagnostic and prognostic biomarker for urothelial cancer. However, further studies are required to confirm its clinical applicability 16.

## Histological and Genetic Biomarkers

The study of histological and immunohistochemical biomarkers in urothelial cancer begins with the collection of tumor tissue samples, primarily through transurethral resection of the bladder (TURB), cystoscopic biopsy, or cystectomy, with TURB being the most common diagnostic method for histological confirmation and staging. To further assess tumor aggressiveness, prognosis, and therapeutic response, immunohistochemistry (IHC) is employed to detect specific antigens in tumor cells through monoclonal or polyclonal antibodies, enabling precise biomarker visualization under a microscope via antigen retrieval, antibody incubation, enzymatic labeling, and chromogenic detection. On the other hand, advances in genomics have enabled the identification of genetic biomarkers in urothelial bladder carcinoma, providing valuable insights into tumor biology and therapeutic implications.

### Histological biomarkers

In the study by Kim et al., several histological and immunohistochemical markers were analyzed 28. Among 118 patients with high-grade non-muscle-invasive bladder cancer (NMIBC) followed for an average of 64.3 months, 15.3% experienced disease progression. The study highlighted the prognostic relevance of E2F1, p27, and the proportion of the invasive component, reinforcing the need to incorporate molecular markers in clinical practice to improve risk stratification and therapeutic strategies.

E2F1 is a transcription factor that plays a crucial role in cell cycle regulation. Its overexpression has been observed in patients with progressive disease, suggesting its involvement in bladder cancer aggressiveness 29. The dysregulation of E2F1 is associated with increased proliferation and impaired apoptotic mechanisms, making it a potential target for therapeutic interventions.

p27, a cyclin-dependent kinase inhibitor, regulates cell cycle progression by preventing transition through the G1 phase. Its tumor suppressor role is particularly relevant in urothelial carcinoma, where loss of p27 expression has been linked to poor prognosis. Rabbani et al. demonstrated that decreased p27 expression correlates with a higher risk of pelvic recurrence, metastatic progression, and mortality in bladder cancer patients 30,31. The reduction in p27 levels is often associated with an increase in cyclin E activity, which drives unchecked cell cycle progression, a hallmark of aggressive tumor phenotypes.

IMP3 is another significant biomarker expressed in muscle-invasive bladder cancer (MIBC). Immunohistochemical detection of IMP3 has proven useful in predicting tumor progression and metastasis, suggesting that its inclusion in diagnostic panels could refine prognosis and guide treatment decisions 32. IMP3 is involved in RNA-binding and post-transcriptional regulation of oncogenes, and its role in epithelial-mesenchymal transition (EMT) further supports its relevance in tumor progression.

A study by Wu et al. identified nine key immunohistochemical markers using a LASSO Cox regression model. These markers include EGFR, HER2, VEGF, CyclinD1, BAX, MDR, TP53, p27, and TOPOII. The combined use of these biomarkers provided higher prognostic accuracy compared to single-marker analysis, potentially improving clinical decision-making and postoperative monitoring 33. This panel reflects the complex interplay of oncogenic signaling pathways in urothelial carcinoma, where alterations in growth factor receptors, cell cycle regulators, and apoptotic mediators contribute to disease progression.

### Genetic biomarkers

Beyond histological and immunohistochemical markers, genetic biomarkers play a crucial role in characterizing bladder cancer at a molecular level. Recent advances in genomic profiling have allowed for a deeper understanding of the mutational landscape of urothelial carcinoma, paving the way for targeted therapies and precision medicine approaches.

FGFR3, a fibroblast growth factor receptor located on chromosome 4, is frequently altered in bladder cancer 34. Its activation triggers dimerization and transphosphorylation of tyrosine residues, leading to downstream signaling via four major pathways: RAS-MAPK, PI3K-AKT, PLCγ, and STAT. Dysregulation of these pathways results in uncontrolled cell growth, proliferation, differentiation, and survival, ultimately contributing to tumor development 35.

In bladder urothelial carcinoma, 15% of cases harbor FGFR3 somatic mutations, 7% show FGFR1 amplification, and 6% exhibit genetic fusions 36. The luminal-papillary subtype of bladder cancer presents FGFR alterations in up to 65% of cases 37. These findings have led to the development of FGFR inhibitors, which have demonstrated efficacy in patients with muscle-invasive urothelial carcinoma who have progressed after platinum-based chemotherapy. Targeting FGFR3 has emerged as a promising therapeutic approach, particularly for patients with mutations or gene fusions affecting this pathway.

p53 is a tumor suppressor gene that plays a central role in cellular response to DNA damage. Loss of tumor suppressor genes such as PTEN has been linked to epithelial senescence in the bladder, functioning as a protective mechanism against tumor formation 38. The miRNA-21-PTEN/p53 axis significantly influences urothelial carcinoma progression by disrupting its interaction with the negative regulator MDM2 39. The dysregulation of p53 is a common event in high-grade bladder cancer, often leading to resistance to conventional therapies.

Aberrant signaling in the mTOR pathway, frequently caused by PTEN downregulation, contributes to tumor progression. This has prompted the investigation of mTOR inhibitors as potential therapeutic agents for muscle-invasive urothelial carcinoma 40. Additionally, increased p53 gene expression is associated with a higher recurrence risk in urothelial carcinoma 41,42. The interplay between p53 and the DNA damage response machinery suggests that combination therapies targeting p53 restoration and checkpoint inhibitors could enhance treatment efficacy.

SPINK1, or serine peptidase inhibitor Kazal type 1, was initially identified in pancreatic acinar cells but is now recognized for its involvement in various malignancies, including breast, ovarian, head and neck, lung, gastrointestinal, and urological cancers 43. SPINK1 is produced by human stromal cells in response to DNA damage and is regulated via the NF-κB and C/EBP signaling pathways 44.

Jiang et al. demonstrated a negative correlation between SPINK1 expression and overall survival in muscle-invasive bladder cancer, in contrast to eight oncogenes (CCDC80, CD3D, CIITA, FN1, GBP4, GNLY, UBD, and VIM) that are positively correlated with one another in this malignancy 45. SPINK1 has also been implicated in resistance to chemotherapy, further underscoring its relevance in clinical decision-making.

**Figure 1:**
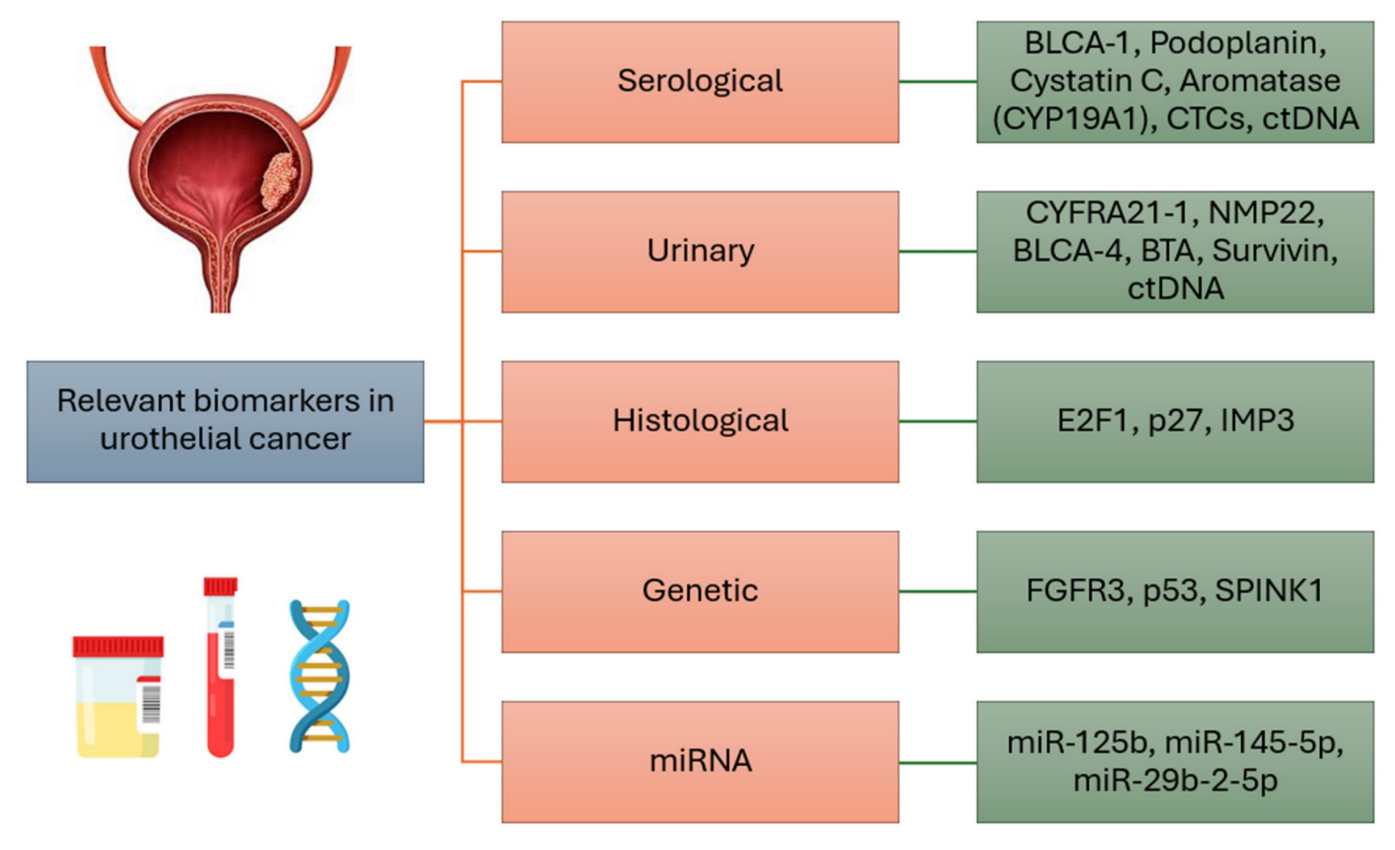
Summary of the most relevant biomarkers in urothelial cancer.

The most relevant serological, urinary, histological and genetic markers, as well as microRNAs of urothelial breast cancer, are concisely summarized in **Figure 1**.

## Role of MicroRNA in urothelial cancer

MicroRNAs (miRNAs) are a class of small ribonucleic acid (RNA) molecules ranging from 20 to 25 nucleotides in length, functioning as key post-transcriptional regulators of gene expression in plant, animal, and viral cells. Since their discovery in 2001, miRNAs have been identified as critical modulators of various cellular processes by negatively regulating gene expression at the post-transcriptional level. This regulation occurs through their binding to the untranslated 3' region (3'UTR) of target mRNA, leading to translation inhibition or mRNA degradation 46.

Using diverse molecular techniques, miRNAs have been detected in multiple cancer types, with some serving as characteristic of different tumors 47. This discovery has paved the way for novel research into their potential applications in cancer diagnosis.

In 2013, Jaime Snowdon et al. conducted a study to evaluate the diagnostic potential of specific miRNAs in urine samples from patients with urothelial carcinoma, aiming to develop a non-invasive detection method for this type of cancer. Urine samples were collected from bladder cancer patients prior to tumor resection, alongside samples from a healthy control group. Total RNA was extracted from these samples, and quantitative real-time PCR (qRT-PCR) analysis was performed to assess the expression of four miRNAs previously identified in urothelial tumors. Notably, significant differences in the expression of two miRNAs were observed in bladder cancer patients. miR-125b exhibited a 10.42-fold reduction compared to healthy controls (p < 0.01), while miR-126 demonstrated a 2.70-fold increase, though without statistical significance (p = 0.30). Both miRNAs achieved 100% specificity and 80% sensitivity for cancer detection, whereas urinary cytology demonstrated a sensitivity of 50% and specificity of 80%. These findings suggest that urine miRNAs could serve as reliable biomarkers for bladder cancer diagnosis, offering improved accuracy over traditional cytology 48.

Another significant study, "Study on Small Non-Coding RNAs in Non-Muscle Invasive Bladder Cancer (NMIBC)," conducted by Jiajia Cai et al., focused on analyzing the expression of small non-coding RNAs (sncRNAs) in NMIBC patients to identify deregulation patterns and their implications in disease pathogenesis and treatment. The study included 107 recently diagnosed NMIBC patients at Luohu District Hospital in Shenzhen. Tumor tissue and adjacent healthy tissue samples were collected and analyzed using next-generation sequencing (NGS) to assess the expression profiles of piRNAs and miRNAs. Differential expression levels of sncRNAs were examined, and their potential functions in immune and cancer-related pathways were analyzed. A total of 319 miRNAs were differentially expressed, primarily located on chromosome 14. Among these, deregulated miRNAs such as hsa-miR-490-5p, hsa-miR-204-3p, and hsa-miR-383-5p were associated with key cancer signaling pathways, including the TNF pathway, apoptosis, and cell proliferation. The study concluded that certain miRNAs could be utilized as biomarkers for diagnosis and prognosis, as well as potential therapeutic targets to enhance immunotherapy strategies. However, further studies are required to validate their clinical applicability and specific roles in NMIBC progression 49.

Upper tract urothelial carcinoma (UTUC) is a rare genitourinary malignancy, comprising 5% to 10% of urothelial tumors. Its management depends on tumor grade and stage, with treatment options ranging from radical nephroureterectomy (RNU) to kidney-sparing procedures in lower-risk cases. Despite surgical intervention, UTUC exhibits high recurrence and mortality rates, underscoring the need for improved risk stratification to optimize postoperative surveillance and treatment. A study by Hao-Lun Luo et al. investigated miRNAs associated with UTUC, highlighting the role of miR-145-5p. The researchers examined its effect on the expression of 5-aminoimidazole-4-carboxamide ribonucleotide formyltransferase/inositol monophosphate cyclohydrolase (ATIC), a gene linked to tumor growth. BFTC909 cell lines were transfected with miR-145-5p mimics to evaluate changes in protein expression via two-dimensional polyacrylamide gel electrophoresis. qRT-PCR and Western blot analyses were used to assess ATIC mRNA and protein levels. The findings demonstrated that miR-145-5p downregulated ATIC expression at the protein level, with elevated ATIC expression correlating with advanced tumor stage, metastasis, recurrence, and poor prognosis in UTUC patients. Furthermore, ATIC inhibition significantly suppressed UTUC cell proliferation, migration, and invasion, suggesting that miR-145-5p directly regulates ATIC's 3'UTR region 50.

Another study by Brendan M. Browne analyzed miRNA expression profiles in UTUC samples to determine their predictive value for tumor grade, muscle invasion, and survival outcomes. RNA was extracted from tumors of 157 patients who underwent RNU at two hospitals, and miRNA expression was assessed via qRT-PCR. Comparisons of miRNA profiles between high- and low-grade tumors, as well as between tumors with and without muscle invasion, were conducted. A model incorporating miR-29b-2-5p, miR-18a-5p, miR-223-3p, and miR-199a-5p achieved 83% sensitivity, 85% specificity, and an area under the curve (AUC) of 0.86 in predicting high-grade tumors. Another classifier, including miR-10b-5p, miR-26a-5p, miR-31-5p, and miR-146b-5p, exhibited 64% sensitivity, 96% specificity, and an AUC of 0.90. Additionally, miR-10a-5p, miR-30c-5p, and miR-10b-5p were identified as the strongest predictors of recurrence-free survival (RFS), while miR-10a-5p, miR-199a-5p, miR-30c-5p, and miR-10b-5p were most associated with overall survival (OS). These findings suggest that miRNA expression profiles can distinguish between high- and low-grade tumors, as well as between muscle-invasive and non-muscle-invasive tumors. Furthermore, specific miRNAs may serve as prognostic biomarkers for recurrence and overall survival, aiding in patient risk stratification and optimizing postoperative treatment strategies 51.

Additional studies have further expanded on the potential of miRNAs as prognostic markers. Veerla et al. analyzed tissue samples from urothelial carcinoma patients and found that miR-222 and miR-125b were highly expressed in muscle-invasive tumors, while miR-452 and miR-452* were overexpressed in tumors with lymph node metastases, highlighting their prognostic significance 52,53. Kriebel et al. investigated miRNA expression in normal and cancerous tissues, as well as serum samples from UTUC patients. Their findings revealed that miR-141 was significantly elevated in serum compared to individuals with non-malignant urological conditions, achieving an ROC curve area of 0.726, with a sensitivity of 70.5% and a specificity of 73.5% for distinguishing UTUC cases 54.

Overall, these findings suggest that miRNAs represent a promising avenue of research as diagnostic and prognostic biomarkers for patients with urothelial carcinoma. Furthermore, miRNAs hold potential as therapeutic targets, paving the way for more personalized and effective treatment strategies 55.

## Circulating Tumor Cells and Circulating Tumor DNA

Circulating Tumor Cells (CTCs) are cancer cells that detach from primary or metastatic tumors and circulate in the bloodstream, playing a crucial role in cancer dissemination. Their study has gained significance in oncology due to their potential as non-invasive biomarkers for diagnosis, prognosis, and treatment monitoring. The detection and characterization of CTCs allow for the assessment of tumor progression, therapy response, relapse risk, and the identification of potential therapeutic targets 56. Similarly, circulating tumor DNA (ctDNA), composed of tumor-derived DNA fragments released into the bloodstream during cell death, contains tumor-specific mutations. This makes ctDNA a valuable tool for liquid biopsy, enabling the detection of genetic alterations in a non-invasive manner 57, 58.

Various techniques have been developed for the detection of CTCs and ctDNA, each with distinct advantages and limitations*.* CellSearch®, the only FDA-approved CTC detection method, has demonstrated inconsistent results in urothelial cancer due to its inability to identify epithelial marker-negative tumor cells 59. In contrast, next-generation sequencing (NGS) of ctDNA provides a broader analysis of genetic mutations, but its sensitivity is contingent on sequencing depth and associated costs. Emerging technologies, such as single-cell sequencing, hold promise for improving the evaluation of tumor heterogeneity and clonal evolution 60, 61. Studies have also been conducted on the use of droplet digital PCR (ddPCR), which is highly sensitive (0.01%) but has a limited capacity to detect multiple alterations 62. Furthermore, an innovative microfluidic device has been developed for the detection of CTCs in bladder cancer 63, 64. This system employs the biotinylated monoclonal antibody BCMab1, designed to specifically recognize aberrantly glycosylated integrin α3β1, a characteristic biomarker of bladder tumor cells. Compared to conventional methods such as flow cytometry and PCR, this technology stands out for its higher precision, lower sample volume requirement, and a simplified detection process. In tests conducted with blood samples from bladder cancer patients, the device achieved a 90% CTC capture rate under optimal conditions. Although the current platform is not yet high-throughput, it could play a role in the future diagnosis and monitoring of bladder cancer 65,66.

Despite advances in urothelial cancer treatment, early detection of minimal residual disease and improved risk stratification remain critical challenges for enhancing survival outcomes and minimizing overtreatment. CTCs and ctDNA have shown potential as complementary liquid biopsy approaches in bladder cancer. In a pilot study of 16 patients with metastatic UC, both methodologies were analyzed to determine their comparative utility. The results showed that 75% of patients had detectable CTCs, while 73% had detectable mutations in ctDNA, with no correlation between the two. Notably, ctDNA analysis identified clinically actionable mutations that were not detected in tumor tissue. Furthermore, a ctDNA fraction >2% was significantly associated with worse overall survival, whereas CTC detection did not show a statistically significant prognostic correlation. These findings suggest that CTCs are useful for studying the biological characteristics of UC, while ctDNA may be more effective for early detection and disease monitoring 67. The combination of both methodologies could optimize risk stratification and therapeutic selection in metastatic UC, further advancing the integration of liquid biopsy into precision oncology.

### Circulating Tumor Cells

In a study involving 100 patients with high-risk non-muscle invasive bladder cancer (NMIBC), who underwent transurethral resection of bladder tumor followed by adjuvant intravesical therapy, CTCs were analyzed prior to the first intravesical therapy. The results indicated that 56 patients were CTC-positive, and these individuals exhibited shorter time to first recurrence (7.1 months vs. 15.5 months in CTC-negative patients, P < 0.001) and shorter time to progression (8.5 months vs. 17.4 months in CTC-negative patients, P < 0.001). These findings suggest that the presence of CTCs is associated with a poorer prognosis. Gene expression analysis in CTCs showed significant differences in tumor progression-related genes. CD133 was exclusive to CTC-positive samples, while KRAS, Survivin, PI3K, and VEGF were overexpressed, and TP53 was downregulated. KRAS, EPCAM, CD133, and Survivin were strongly linked to recurrence and progression, with VEGF and CD44 also elevated in progressive cases 68. These findings highlight CTCs as prognostic biomarkers in NMIBC, with potential to improve risk stratification and guide early radical cystectomy in high-risk patients.

It has been shown that the presence of CTCs is more frequent in patients with metastatic urothelial cancer and has been associated with lymphovascular invasion and positive surgical margins 69. Their presence prior to radical cystectomy has been linked to worse recurrence-free survival and overall survival. Soave et al., in an analysis of 185 patients with MIBC, found that 22% had detectable CTCs before radical cystectomy. CTC-positive patients had a worse prognosis compared to CTC-negative ones 70. This could help predict which patients have more aggressive tumors and allow for the planning of adjuvant treatments and/or closer post-surgical follow-up. Some studies, such as those by Rink et al. and Chalfin et al., suggest that adjuvant chemotherapy may reduce CTCs in the blood, indicating a potential impact on the control of residual disease 71,72.

Regarding its use as a predictive biomarker for immune checkpoint inhibitor treatment, a study analyzed the expression of PD-L1 in CTCs in the blood of patients with advanced urothelial cancer. CTCs were identified in 47.4% of the analyzed samples, and PD-L1 expression was detected in at least one CTC in 63% of the CTC-positive samples. Moreover, heterogeneity in PD-L1 expression was observed both within individual patients and among different patients. Furthermore, the study demonstrated that the presence of CTCs and higher PD-L1 expression in these cells correlated with a higher risk of disease progression and worse overall survival. Additionally, vimentin expression in CTCs was evaluated as a marker of epithelial-mesenchymal transition, being identified in a small percentage of samples. The detection of both vimentin and PD-L1 could provide additional insights into tumor aggressiveness and treatment resistance 73.

### Circulating Tumor DNA

Regarding detection and characterization of ctDNA in Metastatic Urothelial Cancer. Sonpavde et al. utilized a 73-gene panel to detect ctDNA aberrations in 90% of patients with metastatic urothelial cancer. The most frequently observed mutations were TP53 (48%), ARID1A (17%), and PIK3CA (14%), with ctDNA mutations showing a similar pattern to those previously reported in tumor tissue studies 74. Similarly, McGregor et al., using a 62-gene panel, detected ctDNA in 73% of patients. In cases of cisplatin resistance, ctDNA analysis revealed the persistence of ERBB2 and TP53 mutations, along with new alterations in NF1, highlighting its potential for studying treatment resistance 75.

As for monitoring treatment response and recurrence, Birkenkamp-Demtröder et al. evaluated 26 MIBC patients undergoing neoadjuvant chemotherapy and found that ctDNA was detectable in 50% of patients who later relapsed, with a median detection 137 days before clinical recurrence. Additionally, elevated ctDNA levels post-cystectomy were significantly associated with a higher risk of recurrence 76. Patel et al. in a study of 17 MIBC patients receiving neoadjuvant chemotherapy, demonstrated that ctDNA detected before the second chemotherapy cycle predicted recurrence in 83% of cases, with a median lead time of 243 days before radiological confirmation 77.

The study by Cheng et al. underscored the importance of integrating liquid and tissue biopsies. Among 26 patients with metastatic urothelial cancer, plasma mutations were detected in 69%. However, only 20% of patients had identical plasma and tissue mutation profiles. In 40% of cases, mutations detected in plasma were absent in tissue, suggesting tumor evolution and intratumoral heterogeneity 78.

Concerning the detection of ctDNA in urine, it has emerged as a promising tool for the diagnosis and monitoring of bladder cancer due to the direct proximity between the tumor and urine. Studies have shown that urinary ctDNA more accurately reflects the genetic alterations of the tumor than plasma ctDNA, with one study indicating that 92% of the genetic alterations found in the primary tumor are also present in urinary ctDNA 79. This characteristic makes it useful for monitoring residual disease after radical cystectomy, offering a non-invasive option for postoperative follow-up.

The TOMBOLA study is the first clinical trial to use serial ctDNA measurements to guide treatment decisions in bladder cancer. It is an ongoing multicenter study designed to evaluate the use of ctDNA in patients with muscle-invasive bladder cancer treated with neoadjuvant chemotherapy and radical cystectomy. The study protocol establishes postoperative monitoring through serial ctDNA analyses. If a patient tests ctDNA positive, an additional computed tomography scan is performed, and Atezolizumab immunotherapy is initiated, regardless of whether metastases are visible in the imaging. In contrast, ctDNA-negative patients receive immunotherapy only if metastases are later detected in follow-up scans. Preliminary results show that 57% of patients were ctDNA+ after radical cystectomy, and in 75% of cases, detection occurred within the first four months after surgery. Additionally, in 20% of ctDNA+ patients, CT scans confirmed the presence of metastases, with a median lead time of 43 days before they were visible on conventional imaging. On the other hand, only 3% of ctDNA-negative patients developed metastases during follow-up. These findings suggest that ctDNA monitoring is a highly specific tool, allowing for the early identification of patients who could benefit from early immunotherapy, even in the absence of visible metastases 80, 81.

The main urothelial biomarkers, according to type, description, and clinical application, are summarized in **Table 1.**

## Conclusions

Although the role that different biomarkers may play in urothelial carcinoma of the bladder is established, evidence from clinical trials is still needed to routinely include them in clinical practice. These biomarkers can be obtained from a peripheral blood sample or by analyzing tumor tissue using immunohistochemistry.

Urinary biomarkers have a high rate of false positives to be taken into account and this must be clinically correlated with diagnostic tests for their correct interpretation, however, they are used as an adjunct to cystoscopy in those patients who are being monitored. In addition, urinary biomarkers such as survivin are associated with decreased overall survival rates.

It has been shown that urine miRNAs could be used in clinical practice as a biomarker for the diagnosis of urothelial cancer, in addition to the fact that it has been shown that miRNAs could be used as a prognostic factor since they are expressed in muscle-invasive urothelial carcinoma. As for serological biomarkers, for example, BLCA-1 as an important predictor of inflammation favoring tumor angiogenesis and probable cell dysregulation.

FGFR-targeted therapies have shown clinical benefits if patients present alterations at the level of FGFR3, however, it would be necessary to achieve a broader understanding of the position of these drugs within the different current treatment algorithms.

It will be relevant to continue in the search for the participation that can be obtained with ctDNA to identify those patients who have a high risk of recurrence and thus perform an early medical intervention despite not showing suspicion of macroscopic tumor lesion by imaging techniques. Similarly, the detection of urinary ctDNA would also make it possible to monitor, even detecting genetic alterations of the tumour more accurately.

## Figures and Tables

**Table 1:** This table summarizes the clinical applications of specific biomarkers in urothelial cancer, categorized by type, with detailed descriptions and corresponding references for each agent.

Biomarker	Type	Description	Clinical Applications	Reference
BLCA-1	Serological	A protein selectively expressed in bladder cancer cells, detectable in urine and serum.	Potential non-invasive diagnostic biomarker, associated with inflammatory cytokines like VEGF and IL-8.	15, 16
Podoplanin	Serological	A transmembrane glycoprotein involved in EMT, promoting tumor invasiveness and lymphangiogenesis.	Elevated levels in aggressive bladder tumors, with a diagnostic sensitivity of 72% and specificity of 69%.	17, 18
Cystatin C	Serological	Low-molecular-weight protein, endogenous inhibitor of cysteine proteases.	Lower levels in UC patients, suggesting tumor suppression potential.	19
Aromatase (CYP19A1)	Serological	Enzyme responsible for androgen-to-estrogen conversion, influencing tumor microenvironment.	Linked to higher tumor aggressiveness and reduced survival in MIBC.	20, 21
CYFRA21-1	Urinary	A cytokeratin fragment released by urothelial tumor cells.	Elevated urinary levels correlate with metastatic bladder cancer cases.	22
NMP22	Urinary	Nuclear mitotic apparatus protein detected via urine immunofluorescence.	Enhances cystoscopy accuracy; sensitivity ~70%.	23, 24, 25
BLCA-4	Urinary	A nuclear matrix protein excreted in urine.	High sensitivity (89-97.37%) and specificity (90-100%) for high-grade tumors.	26
BTA	Urinary	Bladder Tumor Antigen from basal membrane degradation.	BTA-Stat and BTA-Trak tests reduce false positives in urine.	26, 27
Survivin	Urinary	Apoptosis inhibitor regulating tumor resistance.	Overexpression linked to poor prognosis and reduced survival.	26
E2F1	Histological	Transcription factor crucial for cell cycle regulation.	Overexpression associated with disease progression and aggressive UC.	28, 29
p27	Histological	Cyclin-dependent kinase inhibitor controlling G1 phase progression.	Loss of p27 correlates with poor prognosis and increased metastasis risk.	30, 31
IMP3	Histological	RNA-binding protein involved in oncogene regulation.	Predicts tumor progression and metastasis in MIBC.	32
FGFR3	Genetic	Fibroblast growth factor receptor mutated in UC.	Target for FGFR inhibitors in MIBC therapy.	34, 35, 36, 37
p53	Genetic	Tumor suppressor gene involved in DNA damage response.	Loss linked to increased recurrence risk and therapy resistance.	38, 39, 40
SPINK1	Genetic	Serine peptidase inhibitor with oncogenic activity.	Associated with worse survival outcomes in MIBC.	43, 44, 45
miR-125b	MicroRNA	miRNA downregulated in bladder cancer.	Potential diagnostic biomarker for UC, with 100% specificity.	48
miR-145-5p	MicroRNA	miRNA targeting ATIC genes involved in tumor progression.	Suppresses cell proliferation, migration, and invasion in UTUC.	50
miR-29b-2-5p	MicroRNA	miRNA linked to tumor grade and invasion.	Predicts high-grade tumors with 83% sensitivity, 85% specificity.	51
CTCs	Serological	Circulating tumor cells shed into the bloodstream.	Prognostic marker for tumor progression and treatment response.	56, 57, 58
ctDNA	Serological	Tumor-derived DNA fragments in blood.	Allows for non-invasive detection of genetic mutations.	74, 75, 76
Urinary ctDNA	Urinary	Tumor-derived DNA fragments in urine.	More accurate in detecting tumor genetic alterations than plasma ctDNA.	79
